# Port site intramuscular abscess from retained gallstone post laparoscopic cholecystectomy – an unusual complication

**DOI:** 10.1093/jscr/rjac611

**Published:** 2023-01-04

**Authors:** Faisal Syed, Gaik Si Quah, Angelina Di Re

**Affiliations:** Department of Surgery, Dubbo Base Hospital, Dubbo, New South Wales, Australia; Department of Surgery, Dubbo Base Hospital, Dubbo, New South Wales, Australia; Department of Surgery, Dubbo Base Hospital, Dubbo, New South Wales, Australia

## Abstract

Perforation of the gallbladder and the spillage of gallstones can be a cause of ongoing morbidity and mortality in patients post cholecystectomy. We report on an unusual case of a 58-year-old male who developed a right upper quadrant lump 8 years after laparoscopic cholecystectomy which was eventually determined to be an abscess from a retained gallstone that had become embedded in the musculature of his abdominal wall. We have also discussed some of the considerations taken in the surgical management of this case.

## INTRODUCTION

The spillage of gallstones during cholecystectomy can result in long-term morbidity or mortality if they are not completely retrieved. As it is not possible to predict where a retained gallstone may finally lie, so too is not possible to predict when or how a patient may manifest their subsequent complication. Symptoms may range from simple discomfort or pain, to abscess or collections or even potentially devastating fistulae or sepsis. We present a case of an abdominal wall abscess likely due to a retained gallstone dislodging and embedding into the muscle upon removal of the procedural abdominal drain.

## CLINICAL SCENARIO

A well 58-year-old male presented to the surgical clinic with a palpable small tender lump on his right upper quadrant on a background of a previous emergency laparoscopic cholecystectomy for acute cholecystitis 8 years ago. At the time of his emergency procedure, his observations were normal and blood investigations revealed elevated inflammatory markers (Tables 1 and 2). He had a background of hypertension, hypercholesterolaemia and gout, and no previous surgical history.

**Table 1:** Vital signs at the time of presentation to A + E.

Pulse	65 p/m
Blood pressure	121/61
Temp	35.8
Pain score	5/10
Respiratory rate	18
O_2_ saturation	100%
News score	1

**Table 2:** Bloods investigation at the time of presentation to A + E.

Haemoglobin	136	NR: 130–180 g/L
White cell count	15.6	NR: 3.7–9.5 × 10*9/L
Platelets	289	NR: 150–400 × 10*9/L
Neutrophils	11.9	NR: 2.0–8.0 x 10*9/L
Sodium	138	NR: 135–145 mmol/L
Potassium	4.5	NR: 3.2–5.0 mmol/L
eGFR	>90	NR: 60–90 ml/min
Urea	5.2	NR: 3.0–7.5 mmol/L
AST	14	NR: <40 u/L
ALT	21	NR: <40 u/L
Bilirubin	10	NR: <40 umol/L
CRP	60	NR: <3 mg/L

His initial cholecystectomy was largely uneventful with a single 6 mm immobile gallstone on pre-operative ultrasound and a normal intra-operative cholangiogram without filling defects or anatomical variant. A 10Fr Blake drain was inserted through the 5 mm medial subcostal port site and removed prior to discharge. Histopathology confirmed acute cholecystitis, but no gallstones were identified. An 18 mm disruption to the distal portion where the wall was incomplete was noted suggestive of an iatrogenic perforation.

**Figure 1:**
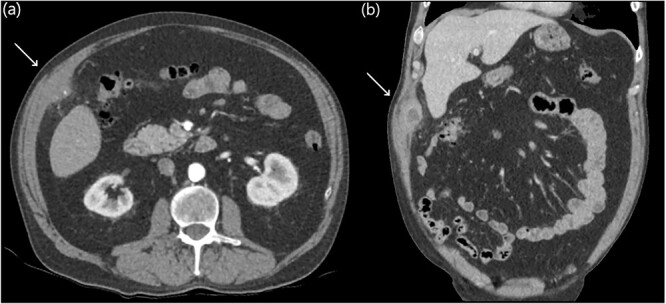
Abdominal CT axial slices (**a**) demonstrating a tiny focal calcific density in the right upper quadrant, and coronal slices (**b**) demonstrating a right upper quadrant intramuscular collection.

Eight years later, a computer tomography (CT) scan revealed a thick-walled collection on the deep aspect of the right anterolateral abdominal wall, adjacent to inferior hepatic segment 5 measuring 61 × 24 × 38 mm with a tiny focal calcific density (Fig. 1).

**Figure 2:**
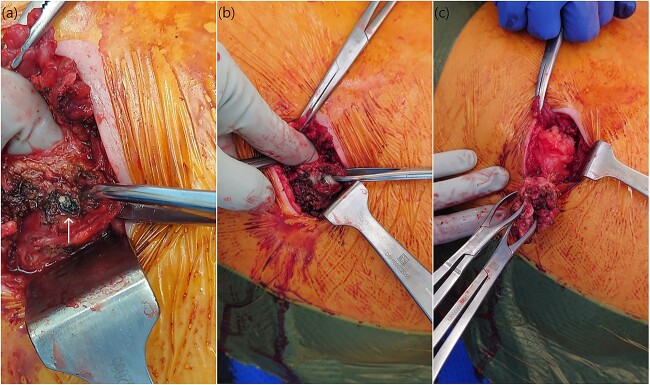
Intra-operative photos demonstrating a small gallstone (**a**) within the muscular tissue revealing the underlying purulent collection (**b**) which was drained with complete excision of the capsule (**c**).

Given the history and imaging findings, we were suspicious of retained gallstones causing abdominal wall abscess. We scheduled the patient for an operative drainage of the anterior abdominal wall collection. On examination, a RUQ lump was palpable and was in close proximity to the medial subcostal laparoscopic port site scar. A chronic intramuscular abscess cavity was found extending to the peritoneum with adherent omentum. Within this abscess cavity, a small gallstone was identified, and the indurated tissue and abscess cavity were excised to reduce the risk of contamination and ongoing infection (Fig. 2). The abdominal wall defect was closed primarily in two layers with 1.0 nylon suture. Histopathology confirmed an abdominal wall abscess with fibroinflammatory tissue and a 5 mm gallstone.

He had an uneventful post-operative recovery and was discharged the next day on a course of oral antibiotics. He was reviewed in clinic ∼3 weeks later; apart from some intermittent post-operative pain at the surgical site on stretching, he had no other complications and his wounds were healing well.

## DISCUSSION

We report on an unusual complication of a retained gallstone resulting in an abdominal wall collection several years after cholecystectomy. It is postulated that the single offending gallstone identified on pre-operative ultrasound was spilt intraoperatively as a result of iatrogenic perforation, eventually became caught in the Blake drain and subsequently dislodged into the muscle layers upon removal, resulting in eventual infection and intramuscular abscess. The incidence of gallbladder perforation has been reported to be within 10–40% with gallstone spillage ranging from 5 to 30% [1, 2]. Multiple case series have demonstrated that spilt gallstones can contribute to long-term morbidity with complication rates between 0.08 and 5% including abscess formation, fistulae and sepsis [1–3]. Complications can have a delayed and variable time-course with symptoms including abdominal pain, palpable masses and sepsis [2, 4, 5].

When faced with calculous cholecystitis, it is ideal to prevent the occurrence of iatrogenic perforation and spillage of gallstones [3, 6]. If spillage occurs it is recommended to attempt retrieval via spoon graspers, retrieval bags or copious peritoneal lavage [3, 5, 7]. Whilst complete retrieval may not always be possible with multiple spilt gallstones; when faced with a minimal number of stones identified on pre-operative imaging, complete gallstone retrieval can be confirmed by ex vivo palpation or examination of the retrieved gallbladder contents. Definitive management of abscess secondary to spilt gallstones typically require abscess drainage and stone retrieval; however, unsuitable patients can be managed with radiologically guided drainage [4, 7].

### Learning points

1. Avoid spillage of gallstones during cholecystectomy.
2. If spillage of gallstones occurs, attempt retrieval of all stones if possible.
3. If there are a low number of gallstones identified on pre-operative imaging, consider confirmation of complete retrieval with ex vivo palpation or examination of the gallbladder specimen.
4. Complications from retained gallstones can present with a variety of signs and symptoms, some of which may be non-specific.
5. Definitive management typically requires surgical retrieval of retained gallstones. In surgically unsuitable patients, source control may be attempted with radiologically guided aspiration or drainage.

## INFORMED CONSENT

The patient has provided informed consent which has been retained by the author’s institution.
